# Multi-Signal Acquisition System for Continuous Blood Pressure Monitoring

**DOI:** 10.3390/s25185910

**Published:** 2025-09-21

**Authors:** Naiwen Zhang, Yu Zhang, Jintao Chen, Shaoxuan Qiu, Jinting Ma, Lihai Tan, Guo Dan

**Affiliations:** 1 School of Biomedical Engineering, Shenzhen University Medical School, Shenzhen University, Shenzhen 518060, China; zhangnaiwen2018@email.szu.edu.cn (N.Z.);; 2 Guangdong-Hongkong-Macau Institute of CNS Regeneration, Ministry of Education CNS Regeneration Collaborative Joint Laboratory, Jinan University, Guangzhou 510632, China; 3 Center for Language and Brain, Shenzhen Institute of Neuroscience, Shenzhen 518057, China

**Keywords:** continuous blood pressure monitoring, multi signal, pulse wave analysis

## Abstract

Continuous blood pressure (BP) monitoring is essential for the early detection and prevention of cardiovascular diseases like hypertension. Recently, interest in continuous BP estimation systems and algorithms has grown. Various physiological signals reflect BP variations from different perspectives, and combining multiple signals can enhance the accuracy of BP measurements. However, research integrating electrocardiogram (ECG), photoplethysmography (PPG), and impedance cardiography (ICG) signals for BP monitoring remains limited, with related technologies still in early development. A major challenge is the increased system complexity associated with acquiring multiple signals simultaneously, along with the difficulty of efficiently extracting and integrating key features for accurate BP estimation. To address this, we developed a BP monitoring system that can synchronously acquire and process ECG, PPG, and ICG signals. Optimizing the circuit design allowed ECG and ICG modules to share electrodes, reducing components and improving compactness. Using this system, we collected 400 min of signals from 40 healthy subjects, yielding 4390 records. Experiments were conducted to evaluate the system’s performance in BP estimation. The results demonstrated that combining pulse wave analysis features with the XGBoost model yielded the most accurate BP predictions. Specifically, the mean absolute error for systolic blood pressure was 3.76 ± 3.98 mmHg, and for diastolic blood pressure, it was 2.71 ± 2.57 mmHg, both of which achieved grade A performance under the BHS standard. These results are comparable to or better than existing studies based on multi-signal methods. These findings suggest that the proposed system offers an efficient and practical solution for BP monitoring.

## 1. Introduction

Hypertension is a widely prevalent chronic disease globally. According to statistics, approximately 63% of Americans and 55% of Chinese individuals aged 45–75 are affected by hypertension [1]. Regular and accurate blood pressure (BP) monitoring is crucial for improving hypertension detection rates and enabling timely intervention and treatment. BP measurement methods can be divided into two categories: invasive and non-invasive. Invasive BP measurement is considered the gold standard, as it provides precise, beat-by-beat BP readings. However, due to its invasive nature and the associated high risk of infection, this method is not suitable for routine monitoring. In contrast, non-invasive methods are more suitable for daily use, with auscultatory and oscillometric techniques being the most commonly employed. These methods, however, require intermittent inflation and deflation of a cuff, which precludes continuous BP monitoring [2]. Additionally, cuff compression can cause patient discomfort. Consequently, there is a pressing need for the development of continuous BP monitoring technologies.

Current research on continuous BP monitoring primarily focuses on technologies based on pulse transit time (PTT) and pulse wave analysis (PWA) [3,4,5,6,7,8,9]. PTT is the time it takes for a pressure wave to travel from a proximal to a distal point along the arterial tree during a single cardiac cycle. Studies have shown a significant linear relationship between BP and the inverse of PTT, which has been widely utilized for BP estimation in numerous studies [10,11,12,13]. Traditionally, PTT is measured using the R-wave peak in the electrocardiogram (ECG) as the proximal reference point, and the peak of the photoplethysmography (PPG) signal or the maximum value of its first derivative as the distal reference point [14]. For example, Xu et al. [15] used the ECG and PPG signals to estimate PTT and applied linear regression methods to predict BP. However, this approach actually measures the pulse arrival time (PAT), not pure PTT [16]. PAT comprises the pre-ejection period (PEP) and PTT, expressed as PAT = PTT + PEP. Therefore, the reliability of using PAT instead of PTT for BP monitoring remains uncertain, as variations in PEP may affect the accuracy of BP estimation.

To eliminate the influence of PEP in PAT, researchers have introduced additional signals to extract a more precise PTT. Among these, impedance cardiography (ICG) is commonly used as an auxiliary signal for PTT acquisition. For example, Finnegan et al. [17] utilized ECG, PPG, and ICG signals to extract PAT, PTT, and PEP. They systematically studied the influence of PEP on PAT and explored the accuracy of BP estimation models based on either PAT or PTT. Their results indicated that both PAT and PTT could be used for BP estimation, with the PTT-based model providing superior prediction accuracy. In contrast, Xie et al. [18] also extracted PAT, PTT, and PEP from ECG, PPG, and ICG signals. They analyzed both linear and nonlinear relationships between BP and these variables, comparing the performance of PTT- and PAT-based models for dynamic BP monitoring. Their findings showed that PTT exhibited a stronger linear correlation with BP, but the PAT-based model generally outperformed the PTT-based model in terms of prediction accuracy. These conflicting results highlight the complexity of BP estimation using these features. Moreover, a limitation of PTT/PAT-based studies is the assumption of a simple linear relationship between PTT/PAT and BP. However, the relationship is actually nonlinear, and the parameters defining this nonlinear relationship can vary between individuals. Therefore, relying solely on PTT/PAT for accurate BP estimation is insufficient.

To enhance BP estimation accuracy, researchers have explored methods based on PWA [19,20]. In PWA-based approaches, BP-related features are typically extracted from various signal waveforms and mapped to BP values using machine learning algorithms. For instance, several PWA parameters extracted from PPG signals, such as systolic rise time, diastolic duration, 2/3 width, and 1/2 pulse amplitude, have been identified as potential indicators for BP estimation [21]. Yang et al. [22] applied PWA method to extract multiple waveform features from ECG and brain bio-impedance signals and then utilized various machine learning algorithms to model these features for BP estimation. They analyzed feature importance, with results indicating that PWA waveform features outperformed PTT features in significance. Furthermore, Yousefian et al. [23] proposed a BP monitoring method that integrates PTT and PWA features. This method collects ballistocardiography (BCG) and PPG signals from the wrist to extract PTT and 36 candidate BCG-PWA features. A specific feature selection algorithm was then applied to identify six features most correlated with BP, which were combined with PTT to form new predictors for BP estimation. The results demonstrated that the BCG-based PTT-PWA fusion technique significantly improved the accuracy and stability of BP monitoring. Currently, PWA-based BP monitoring studies primarily rely on ECG, PPG, and BCG signals [24]. However, compared to these, ICG signals can more directly reflect hemodynamic information, such as cardiac output and vascular resistance, parameters that are closely related to BP changes [25,26]. Nevertheless, research on the integration of ECG, PPG, and ICG signals for BP monitoring remains limited, and the development of relevant technologies and algorithms is still in its early stages.

To address these limitations, this study designs and implements a BP monitoring system based on ECG, PPG, and ICG signals, aiming to achieve continuous BP monitoring. By integrating multiple physiological signals, the system leverages the hemodynamic information from each signal to provide non-invasive BP predictions. During the research, we further investigate the role of PEP in BP prediction, analyzing its potential impact on estimation accuracy. In addition, this study conducted a comparative analysis of the BP monitoring performance of PTT, PAT, and PWA using experimental and statistical methods and provided a comprehensive evaluation of the advantages and limitations of each method. Experimental results show that incorporating the PWA method leads to significant improvements in both prediction accuracy and model stability. By extracting BP-related features from multiple signal waveforms, the PWA method effectively compensates for the limitations of PTT and PAT, bringing the BP estimates closer to the true values. The main contributions of this work can be summarized as follows.

(1) This study develops a compact BP monitoring system that integrates ECG, PPG, and ICG signals. By optimizing circuit design, ECG and ICG modules share electrodes, reducing hardware components and addressing the challenge of increased system complexity.
(2) The system introduces an improved BP estimation approach that integrates multi-signal, effectively filtering noise and extracting key features such as pulse transit time and waveform morphology to enhance signal quality and BP prediction accuracy.

## 2. Materials and Methods

### 2.1. System Overview

**Figure 1:**
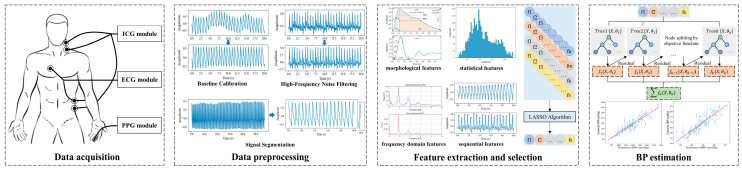
Overview of the BP estimation system.

**Figure 2:**
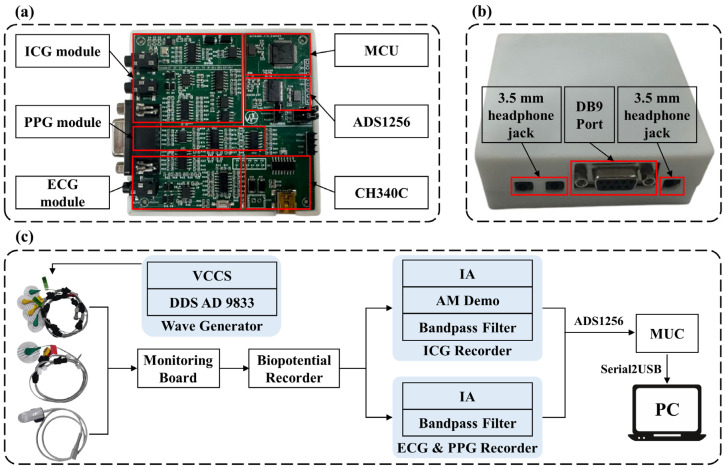
Hardware modules of the BP estimation system. (**a**) System circuit implementation. (**b**) Appearance of the signal acquisition device and external device interface. (**c**) Overall signal acquisition process. IA: Instrumentation Amplifier; DDS: Direct Digital Synthesis; VCCS: Voltage Controlled Current Source; MCU: Microcontroller Unit; AM Demo: Amplitude Modulation Demodulation.

This study proposes a BP monitoring system based on multiple physiological signals, which integrates ICG, PPG, and ECG circuitry units, enabling the simultaneous acquisition and processing of various signals. Figure 1 provides an overview of the BP estimation process with the proposed system. Figure 2a shows the prototype of the multi-signal BP monitoring system board, which consists of six main components: the ICG module, PPG module, ECG module, microcontroller (MCU), analog-to-digital converter (ADS1256, Texas Instruments, Dallas, TX, USA), and USB bus converter chip (CH340C, Nanjing Qinheng, Nanjing, China). The ICG module uses a four-electrode configuration, while the ECG module adopts a lead II configuration, with shared electrodes between the ICG and ECG modules to simplify the hardware design. Additionally, the board integrates a power module and an interface module, featuring standard 3.5 mm headphone jacks for the ECG and ICG interfaces, enabling easy connection to external devices, as shown in Figure 2b. The collected ICG, ECG, and PPG data are transmitted to a PC for further analysis and processing through the MCU’s serial port, with the overall process depicted in Figure 2c. The circuit design for the ICG, PPG, and ECG signal acquisition modules combines the hardware strategy of reliable signal acquisition and the efficient multi-channel data transmission strategy, which is described as follows.

#### 2.1.1. Design of ICG Signal Acquisition Module

The ICG module consists of two main components: the excitation unit and the response unit. The excitation unit generates a constant excitation current, while the response unit detects the chest impedance changes caused by cardiac activity. In the excitation unit, an embedded control system in the lower-level device generates a 50 kHz sine wave signal via a signal generator (AD9833, Analog Devices, Wilmington, MA, USA) and inputs it into a constant current source to produce a sinusoidal current with a root mean square value of 1 mA. This current is then injected into the body through the outer electrodes of a four-electrode lead system, with the inner electrodes used to detect the impedance changes in the chest cavity. Specifically, a high-frequency, low-amplitude constant current is introduced into the chest through the electrode lead system to detect the impedance changes in the thoracic cavity caused by cardiac activity. During systole, blood ejected from the aorta increases thoracic and aortic volumes, leading to a decrease in chest impedance. Conversely, during diastole, reduced blood flow decreases thoracic and aortic volumes, resulting in an increase in chest impedance. In the response unit, the detection circuit amplifies, rectifies, and filters the collected impedance signals. The processed signal is then sampled through the first channel of the ADS1256 (Texas Instruments, Dallas, TX, USA). The acquired ICG signals are transmitted to the MCU for further processing via the Serial Peripheral Interface (SPI) protocol.

It is important to note that the collected signal at this stage represents the voltage variation in the thoracic region, which must be converted into impedance changes using Ohm’s Law. During this conversion, both the overall gain of the ICG acquisition module and the amplitude of the excitation current signal must be considered. The conversion formula is as follows:

(1) $$R_{heart}=\frac{V_{out}}{A\times I_{peak}},$$

where $V_{out}$ is the output ICG voltage signal from the final stage, A is the overall gain of the ICG acquisition module, $I_{peak}$ is the amplitude of the excitation current, and $R_{heart}$ is the impedance change in ohms corresponding to the ICG signal.

**Figure 3:**
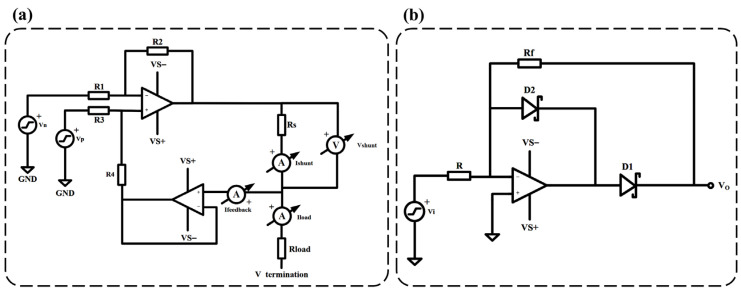
ICG module key circuit diagram. (**a**) Improved Howland current source circuit diagram, R1 = 10 kΩ, R2 = 10 kΩ, R3 = 10 kΩ, R4 = 10 kΩ, and Rs = 200 Ω. (**b**) Precision half-wave rectifier circuit diagram, R = Rf = 750 Ω.

The constant current source circuit and the precision half-wave rectifier circuit are the core components of the ICG measurement module. Figure 3a shows the constant current source circuit of the ICG module, which is designed based on an improved version of the Howland current source. This modified circuit employs two operational amplifiers, optimizing the performance of the traditional Howland current pump. Compared to the conventional design, the improved Howland current pump provides higher output impedance, effectively eliminating the impact of $I_{feedback}$ on $I_{load}$ as shown in Figure 3a, significantly reducing errors. The calculation formula for the output current $I_{load}$ is given by (2), while the formula for the current gain of the output current is given by (3).

(2) $$I_{load}\left(A\right)=\frac{G\times \left(V_{p}-V_{n}\right)}{R_{s}},$$

(3) $$G=\frac{R2}{R1},\;\;when\;R1=R3\;and\;R2=R4,$$

Figure 3b illustrates the precision half-wave rectifier circuit diagram of the ICG module. The primary advantage of using a precision half-wave rectifier design is that it can accurately perform the rectification process even when the input signal voltage is relatively low, without introducing distortion due to the voltage drop across the diode. This precision rectification design effectively preserves the subtle variations in the heart impedance signal, ensuring high-accuracy detection. The expected gain of the half-wave rectifier is given by (4).

(4) $$Gain=-\frac{R_{f}}{R},$$

By selecting an appropriate resistor value, the resistor noise can be made negligible in comparison to the operational amplifier’s voltage broadband noise, as demonstrated in (5).

(5) $$R_{eq}\leq \frac{E_{nbb}^{2}}{4\times k_{b}\times T\times 3^{2}},$$

where $R_{eq}$ represents the parallel combination of resistors R and $R_{f}$. $E_{nbb}$ represents the input voltage noise density of the amplifier, with units of $nV/\sqrt{Hz}$. $k_{b}$ represents the Boltzmann constant, with a value of $1.381\times 10^{-23}\;J/K$. T represents the absolute temperature (in Kelvin, K), assuming room temperature is 298 K.

#### 2.1.2. Design of PPG Signal Acquisition Module

The PPG module uses the DS100-A (Medtronic, Minneapolis, MN, USA) as the acquisition device, which is connected to the circuit board via the DB9 port. This module collects the pulse wave signal from the fingertip using transmission-based measurement, with a red LED serving as the light source. The red light emitted by the LED passes through the fingertip skin and is detected by an integrated photodetector, which converts the light into an electrical signal. After filtering and amplification by the PPG signal conditioning circuit, the electrical signal is sampled through the second channel of the ADS1256. The processed PPG signal is then transmitted to the MCU via the SPI protocol. The PPG signal conditioning circuit includes components such as an I/V conversion circuit, a differential amplifier, an LED driver, and a band-pass filter. We describe the PPG signal conditioning circuit in detail in Supplementary Material S1.

#### 2.1.3. Design of ECG Signal Acquisition Module

The ECG module utilizes a Lead II configuration to acquire ECG signal through disposable silver/silver chloride electrodes. The electrode signals are processed by the ECG signal conditioning circuit, which filters and amplifies the signal before it is sampled through the third channel of the ADS1256. The acquired ECG signal is then transmitted to the MCU via the SPI protocol. We describe the ECG signal conditioning circuit in detail in Supplementary Material S1.

#### 2.1.4. Hardware Strategies for Reliable Signal Acquisition

In wearable devices, ECG, PPG, and ICG signals are susceptible to noise interference, motion artifacts, and crosstalk between modules, which degrade signal quality. To ensure reliable signal acquisition in a compact design, hardware strategies including physical isolation of front-end circuits (at least 10 mm PCB spacing and ground shielding), high-conductivity gel electrodes, and analog-digital separation (using independent power domains and impedance matching) were implemented to minimize crosstalk, motion artifacts, and power line interference. The ADS1256 synchronously samples the signals at 250 Hz with precise timing. In addition, a cascaded second-order Butterworth filter is designed to suppress baseline wander and high-frequency noise, while a right-leg drive circuit enhances the common-mode rejection ratio of the ECG. The fit of the device is optimized with a flexible skin-adhesive material to further reduce motion artifacts. A detailed description of these strategies is provided in Supplementary Materials S2 and S3.

#### 2.1.5. Strategies for Efficient Multi-Channel Data Transfer

To ensure reliable data transmission of ECG, PPG, and ICG modules, the system uses ADS1256 for synchronous signal acquisition at a frequency of 250 Hz, and transmits 24-bit samples to MCU via SPI (10 MHz clock) at a total data rate of 18 kbps, and then transmits frames to PC via serial port (115,200 bps). The 512-byte ring buffer in the MCU prevents data loss, while the power supply design of the ADS1256 ensures signal integrity. We describe the data transmission implementation in detail in Supplementary Material S4.

### 2.2. Data Preprocessing

The quality of ECG, PPG, and ICG signals is primarily affected by three factors: (1) baseline drift caused by low-frequency respiratory motion; (2) power-line interference; and (3) high-frequency noise caused by body movements and muscle contractions.

#### 2.2.1. Baseline Calibration

Among the signals collected in this study, only the PPG and ICG signals exhibit baseline drift, while the ECG signal remains unaffected. A Butterworth high-pass filter is utilized to denoise the signals. In this study, order and cutoff frequency are set to 5 and 0.5 Hz, respectively.

#### 2.2.2. High-Frequency Noise Filtering

High-frequency noise in the signals is primarily caused by muscle tremors and power-line interference. In the collected data, the ICG signal shows noticeable high-frequency noise, while the ECG signal has minimal high-frequency noise, and the PPG signal is free from high-frequency noise. To filter out high-frequency noise in the ICG signal, a Butterworth low-pass filter with a cutoff frequency of 10 Hz and an order of 15 is used. For the ECG signal, a Butterworth low-pass filter with a cutoff frequency of 40 Hz and an order of 15 is applied to remove high-frequency noise. The selection of filter and parameter settings was guided by both existing literature and the characteristics of the collected data [27,28]. The goal was to preserve essential physiological information while effectively eliminating noise.

#### 2.2.3. Segmentation

In this study, a sliding window method is used for data segmentation. This step divides the signals into fixed-length segments to facilitate subsequent feature extraction. A 10 s fixed time window with a 5 s sliding step size is applied, generating overlapping data segments for analysis. This segmentation approach is commonly used in BP monitoring studies [29].

### 2.3. Feature Extraction and Selection

**Figure 4:**
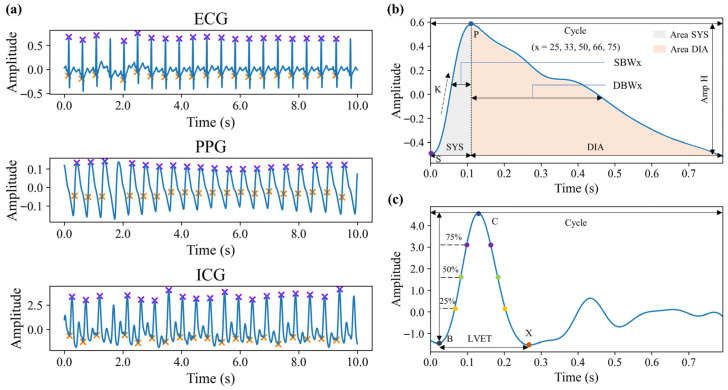
Schematic diagram of signal feature points and morphological features. (**a**) Feature points of three signals. (**b**) Morphological features of PPG signals in a single cardiac cycle. (**c**) Morphological features of ICG signals in a single cardiac cycle.

Before feature extraction, it is essential to identify key points within each 10 s data segment, focusing primarily on the key features of each cardiac cycle. The key points to be identified include: the Q and R points of the ECG signal, the peak and maximum value of the first derivative of the PPG signal, and the B and C points of the ICG signal. Figure 4a illustrates the localization of these key points for each signal. Using these points, the parameters of PTT, PAT, and PEP can be calculated for each cardiac cycle. PAT is defined as the time interval between the Q point of the ECG signal and the peak of the first derivative of the PPG signal. PEP refers to the time interval between the Q point of the ECG signal and the B point of the ICG signal. PTT is the time difference between the B point of the ICG signal and the peak of the first derivative of the PPG signal, i.e., the difference between PAT and PEP.

In addition to traditional BP-related features such as PAT, this study also extracts various PWA features from the PPG and ICG signals. Specifically, morphological features, statistical features, sequential features, and frequency-domain features are extracted from the 10 s segmented signals.

Morphological features are extracted based on individual cardiac cycles. For the PPG signal, cardiac cycles are delineated using valley detection, while for the ICG signal, cardiac cycles are defined based on the R peaks of the ECG signal. The features for each cycle are then averaged to obtain the final feature values. These morphological features include a variety of definitions commonly found in existing research, primarily comprising time-domain parameters, signal amplitudes, area parameters, and their respective ratio coefficients [30,31]. Figure 4b,c visually illustrate these morphological features. Some of the features are defined as follows:

(6) $$K=\frac{P_{a}-S_{a}}{P_{t}-S_{t}},$$

(7) $$SYS=P_{t}-S_{t},$$

(8) DIA=Cycle−SYS,

(9) $$LVET=X_{t}-B_{t},$$

where $P_{a}$, $S_{a}$, $P_{t}$, $S_{t}$, and Cycle represent the amplitude of P and S in PPG signal, the time of P and S, and the time of a cardiac cycle, respectively. $X_{t}$ and $B_{t}$ represent the time of X and B in ICG signal, respectively.

Statistical features are similar to morphological features in that they are also extracted based on individual cardiac cycles and averaged. These statistical features include the mean, median, standard deviation, variance, maximum, minimum, the difference between maximum and minimum, skewness, and kurtosis of the signal.

Sequential features, unlike the previous two types, are extracted from the entire 10 s signal segment rather than from individual cardiac cycles. These features capture the dynamic variation patterns of the signal sequence. Specifically, the HeartPy [32] heart rate analysis toolkit is employed to compute the variability of the signal, including the variability of inter-beat intervals, as well as short-term and long-term scatter standard deviations in the Poincaré plot. Additionally, sample entropy and approximate entropy of the signal sequence are computed to capture the nonlinear dynamic characteristics of the signal.

Frequency-domain features, like sequential features, are extracted from 10 s signal segments. These features are derived from the amplitude spectrum obtained through the Fast Fourier Transform and capture the spectral characteristics of the signal. The extracted features include spectral peak amplitudes and frequencies, spectral energy distribution, and a measure of signal irregularity.

Finally, 88 features are extracted from the PPG signal, and 66 features from the ICG signal in each 10 s segment, resulting in 154 multi-signal PWA features in total. A comprehensive summary and description of the features extracted from PPG and ICG signals are provided in Supplementary Tables S7 and S8.

Although the features extracted from PPG and ICG signals contain valuable information related to BP variations, the original feature set may include redundant or irrelevant features. Using all features directly in model construction can degrade model performance. Feature selection is therefore a crucial step in machine learning workflows, as it can significantly enhance both model efficiency and accuracy. In this study, we employed the Least Absolute Shrinkage and Selection Operator (LASSO) regression algorithm for feature selection [33]. The regularization hyperparameter α was set to 0.1, a commonly used value in similar applications. LASSO introduces an L1-norm penalty to the regression model, which not only minimizes prediction error but also performs variable selection by shrinking some coefficients to zero. This allows for effective feature filtering and model simplification. Using LASSO, we retained only the features most relevant to BP. The selected features are listed in Supplementary Material S5.

### 2.4. Model Development

This study develops five BP monitoring models using the extracted PAT, PTT, and PWA features to estimate BP, providing a comprehensive evaluation of the designed BP monitoring system. The designs of the models are as follows:

Model 1 (PAT): This model utilizes only the PAT feature to construct a univariate linear regression model. It estimates BP through a simple linear regression equation, aiming to assess the contribution of the PAT feature to BP prediction.

Model 2 (PTT): Similar to Model 1, this model uses only the PTT feature to build a univariate linear regression model. It analyzes the effect of the PTT feature on BP prediction. By comparing Model 1 and Model 2, the relative contributions of PAT and PTT to BP monitoring can be evaluated.

Model 3 (PPG-PWA): This is a nonlinear regression model based on PWA features extracted from the PPG. Using machine learning methods, this model is constructed with the XGBoost algorithm, a popular ensemble learning method that improves the prediction model iteratively by adding new weak learners to correct errors. Model 3 is designed to be compared with the linear models to assess the advantages of using nonlinear PWA-based methods for BP prediction.

Model 4 (ICG-PWA): Similar to Model 3, this nonlinear regression model is based on PWA features, but it uses features extracted from the ICG. Like Model 3, it employs machine learning methods and XGBoost for BP prediction.

Model 5 (MS-PWA): This model also uses XGBoost and PWA features for nonlinear regression but incorporates fused features from both the PPG and ICG signals (MS-PWA). The goal of this model is to evaluate the effect of multi-signal fusion on BP monitoring, investigating whether integrating physiological data from different signal sources enhances prediction accuracy. This model further demonstrates the effectiveness and necessity of the proposed system.

Through the design and comparison of these five models, the study systematically evaluates the performance of different features and modeling approaches in BP prediction.

## 3. Experiments

### 3.1. Data Acquisition

This study was approved by the Medical Ethics Committee of Shenzhen University Medical School (approval number: PN-202400147), and all participants have signed informed consent forms. Using the BP monitoring system developed in this study, we collected ECG, PPG, and ICG signals, and corresponding BP data from 40 healthy participants. The participants included 24 males and 16 females, with an average age of 23.9 ± 1.3 years.

**Figure 5:**
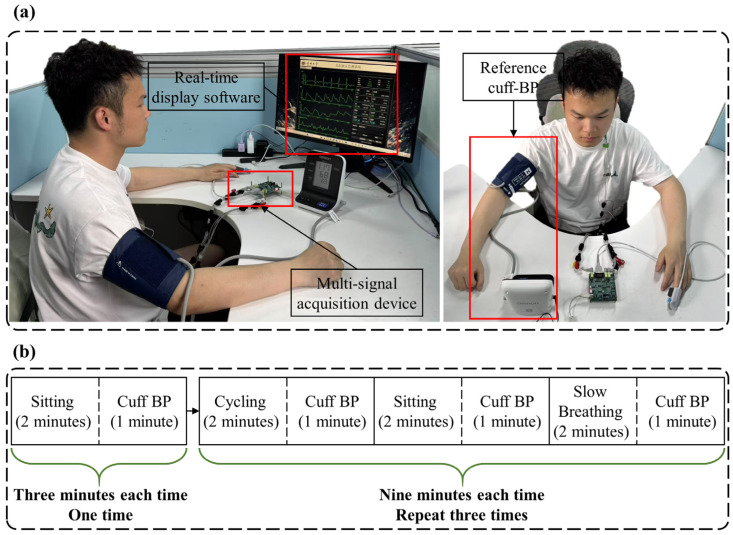
Schematic diagram of data collection. (**a**) Data collection scene of a subject. (**b**) Detailed process of data collection.

Prior to data collection, participants were instructed to refrain from consuming alcohol, coffee, and other stimulating beverages for three days to ensure they were in a well-rested state. Figure 5a shows the data collection scene of a subject. Electrodes for ECG and ICG signal acquisition were placed at the appropriate locations on the body, while the PPG sensor was positioned on the left middle finger. An electronic sphygmomanometer (HBP-1120, Omron, Kyoto, Japan) cuff was worn on the subject’s right upper arm to measure standard BP. The data collection equipment was powered on, the data acquisition software interface was opened, and the start button was pressed to begin automatic data collection. Data were collected in both a resting state and post-exercise. Participants engaged in moderate-intensity cycling on a stationary bike.

The data collection process is detailed in Figure 5b. After the participant donned the equipment, the first stage involved sitting quietly for two minutes, followed by one minute of BP measurement. In the second stage, participants followed these steps: (1) two minutes of cycling to elevate BP, followed by one minute of BP measurement; (2) two minutes of sitting quietly to gradually lower BP, followed by one minute of BP measurement; (3) two minutes of slow breathing (6 breaths per minute) to further reduce BP, followed by another minute of BP measurement. The procedures for the third and fourth stages were identical to those in the second stage. In total, 30 min of signal data and 10 BP measurements were collected from each participant.

**Figure 6:**
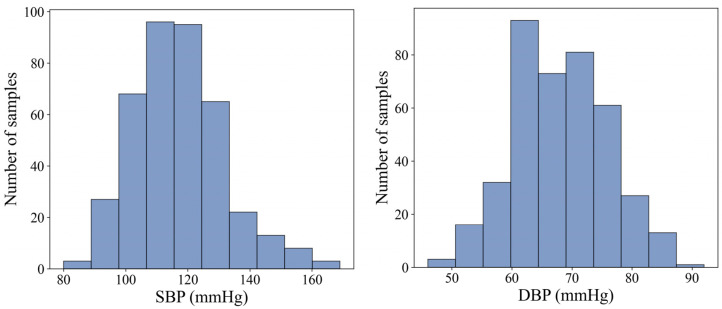
Statistical distribution of SBP and DBP in the acquired data.

Figure 6 illustrates the distribution of the measured reference BP values. During each BP measurement phase, the one-minute BP data were aligned with the corresponding physiological signals. The one-minute signal data were then divided into fixed 10 s windows with a 5 s sliding step, resulting in 11 distinct 10 s segments per minute. Each segment was associated with the reference BP value for that minute. As a result, a total of 4390 valid Signal–BP pairs were obtained.

### 3.2. Evaluation Metrics

Due to the subject-specific nature of PAT/PTT-based BP monitoring methods, the models developed in this study were designed as personalized models for each individual to facilitate comparisons. Specifically, data from the first three phases of each subject were used as the training set to build the model, while data from the fourth phase served as the test set to evaluate model performance. The evaluation of regression models using appropriate metrics is crucial for assessing the accuracy of BP estimation. In this study, model performance was assessed using the following metrics: Mean Absolute Error (MAE), Mean Error (ME), Standard Deviation of Error (SDE), and Pearson Correlation Coefficient (PCC). The definitions of these evaluation indicators are shown in Formulas (10)–(13). In general, lower values of MAE, ME, and SDE indicate better performance, while a higher PCC is preferable. In clinical settings, an MAE below 5 mmHg and an SDE below 8 mmHg are considered acceptable for BP estimation. An ME close to 0 mmHg is ideal, as it suggests the model’s predictions are free from systematic bias. PCC quantifies the strength of the linear relationship between estimated and actual BP values. PCC between 0.5 and 0.7 represents a moderate positive correlation, while a PCC between 0.7 and 0.9 indicates a high positive correlation. To further examine the consistency between estimated and actual BP values, Bland–Altman plots were generated [34]. The Bland–Altman plot is a statistical tool used to assess the agreement between two measurement methods. It visually represents the average difference and the 95% limits of agreement, providing insight into systematic error and variability. A narrower limit indicates higher accuracy, while a wider limit suggests greater differences between the methods.

(10) $$MAE=\frac{1}{N}\sum_{i=1}^{N}\left|X_{i}-Y_{i}\right|\;,$$

(11) $$ME=\frac{1}{N}\sum_{i=1}^{N}\left(X_{i}-Y_{i}\right),$$

(12) $$SDE=\sqrt{\frac{1}{N}\sum_{i=1}^{N}{\left(\left(X_{i}-Y_{i}\right)-E\left(X-Y\right)\right)}^{2}},$$

(13) $$PCC=\frac{\sum_{i=1}^{N}\left(X_{i}-\bar{X}\right)\left(Y_{i}-\bar{Y}\right)}{\sqrt{\sum_{i=1}^{N}{\left(X_{i}-\bar{X}\right)}^{2}}\sqrt{\sum_{i=1}^{N}{\left(Y_{i}-\bar{Y}\right)}^{2}}}\;,$$

Here, N represents the sample size, $X_{i}$ is the predicted value, and $Y_{i}$ is the actual value. $\bar{X}$ and $\bar{Y}$ are the mean values of the predicted and actual values, respectively.

## 4. Results

### 4.1. Signal Quality Assessment

Before analyzing the BP prediction results, we first evaluated the quality of the ECG, PPG, and ICG signals to ensure the reliability of the input signals for the subsequent BP prediction models. The quality of the signals was assessed using the Signal-to-Noise Ratio (SNR).

**Figure 7:**
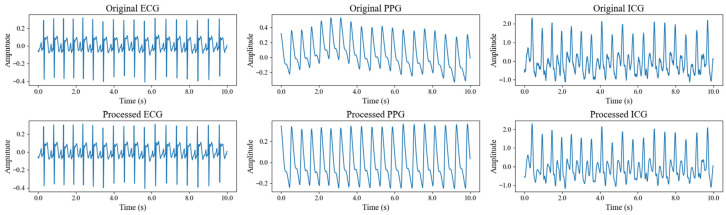
Comparison of three signals of a subject before and after signal processing.

The calculation results revealed that, among the 40 subjects, the average SNR was 29.98 dB for the ECG signal, 8.69 dB for the PPG signal, and 11.44 dB for the ICG signal. These values indicate that the ECG and ICG signals exhibited relatively high quality, while the PPG signal showed lower quality. Figure 7 compares the three signals from a single subject, before and after processing, clearly illustrating the improvement in signal quality. It is important to note that baseline drift and motion artifacts are two major factors that degrade signal quality during acquisition. Baseline drift, primarily caused by respiratory activity, introduces low-frequency noise—particularly in PPG signals—and significantly contributes to their low SNR. Motion artifacts, typically resulting from sudden body movements, generate high-frequency noise across all signal types, with the most pronounced effects observed in ICG signals, followed by ECG and PPG signals. To mitigate these issues, participants were instructed to breathe steadily and minimize movement during data collection. Additionally, the denoising method employed in this study effectively suppresses noise, ensuring the signal quality necessary for reliable feature extraction. Overall, both the SNR analysis and visual comparison confirm that all three signals achieved high quality after filtering, making them ready for BP prediction. In addition, key feature points were located based on the processed signals, and the algorithmic outputs were compared with manual annotations through visual inspection. The results showed that R-wave detection in ECG signals was almost error-free, while the localization error for the first-derivative peaks in PPG signals was generally within five sampling points. The detection of the C wave in ICG signals also demonstrated high reliability. Overall, the detection error rate for all key feature points remained below 2%.

### 4.2. Performance of BP Estimation

Table 1 presents the BP prediction performance of models based on various features, including linear regression models using PAT and PTT features, and nonlinear regression models based on PWA features derived from PPG, ICG, and multi-signal fusion. The results demonstrate that all models perform effectively in monitoring BP, highlighting the efficacy of the multi-signal-based BP monitoring system proposed in this study. Notably, nonlinear regression models based on PWA features outperformed linear models based on PAT and PTT features, with the MS-PWA model (which fuses multiple signal features) achieving the best predictive performance.

In the linear models, the PAT-based model outperformed the PTT-based model, with an MAE of 5.75 for SBP and 3.92 for DBP in the PAT model, compared to an MAE of 8.27 for SBP and 4.18 for DBP in the PTT-based model. This suggests that PEP significantly contributes to BP prediction in this dataset. In the nonlinear models, the PWA model based on PPG signals outperformed the ICG signal-based model, achieving an MAE of 3.95 for SBP and 2.76 for DBP, while the ICG signal model had an MAE of 4.22 for SBP and 3.04 for DBP. The MS-PWA feature model exhibited the best performance, with an MAE of 3.76 for SBP and 2.71 for DBP, outperforming all other models. To further evaluate the generalization ability of the proposed system, in addition to the subject-specific split, we also performed subject-independent validation using five-fold cross-validation. The specific results are in Appendix A.

In summary, PWA features provide more effective BP prediction than single PAT/PTT features, due to the inherent limitations of PAT and PTT in capturing the full complexity of BP dynamics. PWA features, by contrast, capture BP information from multiple perspectives, enabling complementary predictive performance across different features. Moreover, the superior performance of the MS-PWA model, compared to the PPG-PWA and ICG-PWA models, can be attributed to the fusion of multiple signal features, which provides a more comprehensive and accurate representation of vascular characteristics and BP, thus enhancing prediction accuracy.

**Figure 8:**
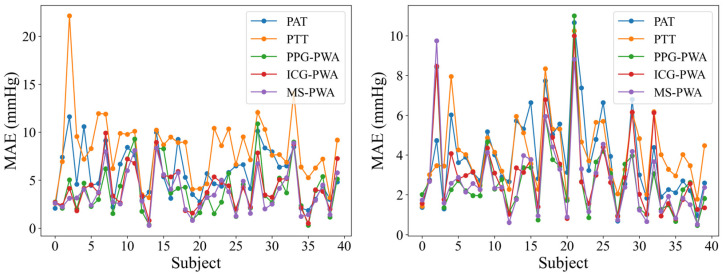
Line chart of MAE indicators for BP prediction using different models.

Figure 8 shows the line plots of the MAE for BP prediction across five models in 40 subjects. The trend indicates that the MAE for most subjects is below 10 mmHg, suggesting that the prediction errors for these models generally remain at a relatively low level, demonstrating reliable predictive performance. The error curves for each model are closely aligned for the majority of subjects, indicating a certain level of consistency in their BP predictions. However, despite the general consistency across the five models, there are still noticeable differences in error between them. Among the models, the MS-PWA model stands out. Compared to the other models, the MS-PWA model exhibits lower error values and smoother curves across all subjects, indicating higher stability in BP prediction.

**Figure 9:**
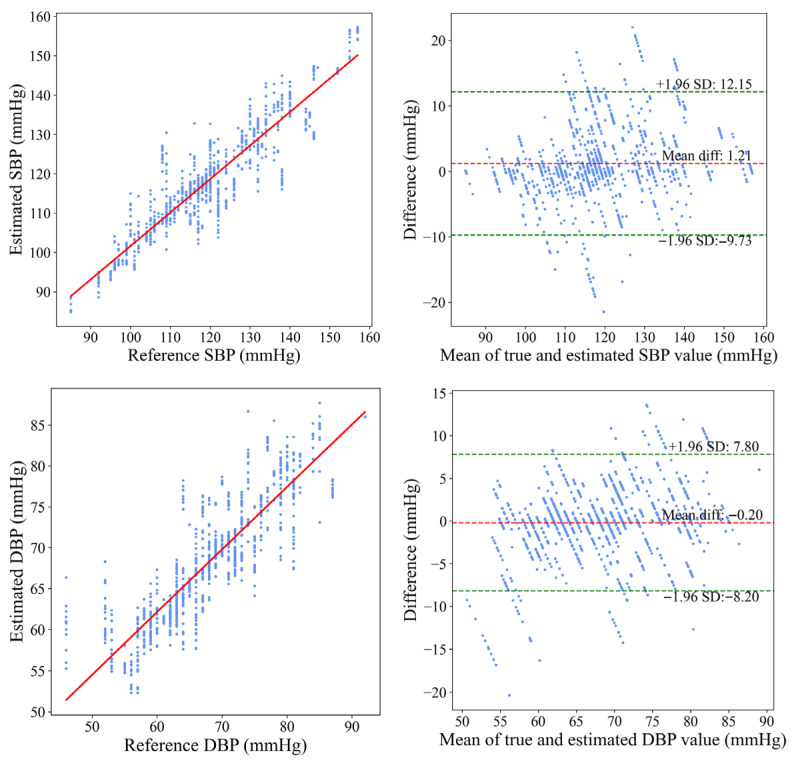
Bland–Altman and correlation plots of SBP and DBP with reference BP.

To clearly illustrate the relationship between the MS-PWA model and the cuff-based reference BP values, Figure 9 presents both a correlation plot and a Bland–Altman plot comparing the MS-PWA predicted BP with the reference values. The correlation plot (left panel of Figure 9) shows a dense clustering of data points along the line Y = X, indicating a strong linear correlation between the predicted and reference BPs. These results demonstrate that both SBP and DBP predictions closely align with the reference values, with most data points near the ideal reference line. The Bland–Altman plot (right panel of Figure 9) visually represents the measurement bias and consistency between predicted and reference values. The mean error for SBP was 1.21 mmHg (95% limits of agreement (LoA): −9.73 to 12.15 mmHg), and for DBP, it was −0.20 mmHg (95% LoA: −8.20 to 7.80 mmHg). These results indicate that the BP estimation method of the MS-PWA model exhibits no significant systematic deviation from the reference method. Additionally, the narrow 95% LoA range, with most data points falling within this interval, further confirms the high measurement consistency of the proposed method. Overall, these results validate the accuracy and reliability of the MS-PWA model for BP prediction.

To further validate the practical applicability of the proposed model, we compared its performance against a well-recognized BP measurement validation standard: the British Hypertension Society (BHS) standard. According to the BHS standard, the cumulative error percentage for all estimated samples was calculated at error thresholds of 5, 10, and 15 mmHg, classifying the model into four grades (A to D). As shown in Table 2, the MS-PWA model achieved the highest grade (Grade A) for both SBP and DBP predictions, further demonstrating its accuracy and reliability. These findings demonstrate that the MS-PWA model delivers excellent predictive performance in the BHS standard, providing strong evidence for its accuracy in BP measurement.

## 5. Discussion

### 5.1. System Effectiveness

Continuous BP monitoring is a valuable tool for assessing an individual’s health and identifying potential underlying conditions. Different physiological signals offer unique advantages in BP measurement. This study presents a multi-signal-based BP monitoring system that synchronously collects ECG, PPG, and ICG signals to enable precise monitoring. By applying both linear and nonlinear models to traditional BP features (such as PAT and PTT) and features derived from PWA, we achieved exceptional BP prediction performance, demonstrating the effectiveness of the proposed system.

This study presents a multi-signal acquisition system that integrates circuit units for ICG, PPG, and ECG, enabling the simultaneous collection and processing of all three physiological signals. By optimizing circuit design, ECG and ICG modules share electrodes, reducing hardware components and addressing the challenge of increased system complexity. Additionally, a user-friendly interface was developed to facilitate real-time monitoring of the collected signals, allowing for immediate assessment of signal quality. A comparison of the signal waveforms before and after filtering, along with the calculation of the SNR, demonstrated that after filtering, all three signals met the quality standards required for accurate BP prediction. Furthermore, the system incorporates a BP estimation method that effectively fuses key features from multiple signals, resulting in improved accuracy in BP prediction.

To validate the effectiveness of the BP monitoring system, we conducted experiments in which traditional BP features (PAT and PTT) were extracted from the ECG, PPG, and ICG signals, and linear regression models were used to predict BP based on these features. Additionally, PWA features were extracted from the PPG and ICG signals, and the XGBoost algorithm was applied to predict BP using PPG-PWA, ICG-PWA, and fused MS-PWA features. The results demonstrated that the MS-PWA model achieved the best performance. In experiments with 40 subjects, the MAE for SBP was 3.76 mmHg and for DBP was 2.71 mmHg, both of which met the BHS standard. These findings confirm that the multi-signal BP monitoring system can collect high-quality signals and provide accurate BP predictions. Its compact design makes it suitable for integration into wearable devices for continuous BP monitoring. In particular, it serves as a valuable supplementary tool for hypertensive patients, addressing the current challenge of achieving continuous BP monitoring in daily life.

### 5.2. The Role of Pre-Ejection Period

**Figure 10:**
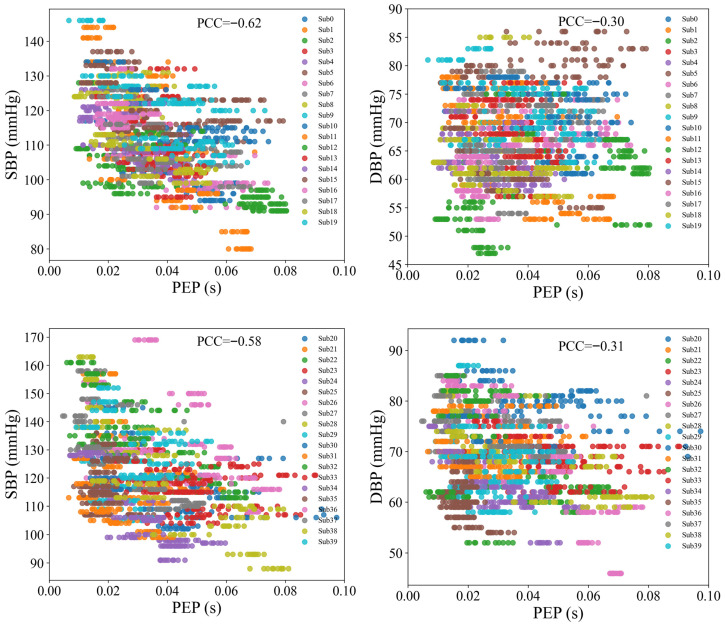
Scatter plots of PEP with SBP and DBP.

PEP is the time interval between the onset of ventricular contraction and the opening of the aortic valve, corresponding to the period from the start of the QRS complex in the ECG signal to the initiation of blood ejection. The relationship between PEP and BP has been widely debated, with varying findings across studies [18,35,36]. Figure 10 shows the scatter plot of PEP and BP in this study, revealing a negative correlation between the two. PEP exhibits a stronger negative correlation with SBP, with a Pearson correlation coefficient of −0.60, while the correlation with DBP is weaker, at −0.30. Table 1 compares the BP prediction performance based on PAT and PTT features. The results show that using the PAT feature yields better prediction accuracy than the PTT feature, indicating that PEP positively contributes to BP estimation. This effect is particularly pronounced in SBP prediction, where the MAE for PAT is 5.75 mmHg, significantly lower than the MAE for PTT (8.27 mmHg). In contrast, the improvement in DBP prediction is smaller, with the MAE for PAT at 3.92 mmHg, slightly better than the MAE for PTT (4.18 mmHg). These results underscore that PEP has a more substantial impact on SBP estimation, while its influence on DBP estimation is comparatively modest. Overall, the findings highlight the crucial role of PEP in enhancing the accuracy of BP estimation, especially for SBP.

### 5.3. Comparison with Previous Studies

Table 3 summarizes recent studies on continuous BP measurement using multiple signals, highlighting differences in signal types and model performance. Many of these studies have employed PTT or PWA techniques for BP estimation. For instance, Ganti et al. [37] combined single-lead ECG, three-axis seismocardiography (SCG), and multi-wavelength PPG signals to measure PTT for continuous BP monitoring. Similarly, Kim et al. [38] used PTT calculated from PPG and ECG signals, alongside features like the I-J interval and J-K amplitude from Ballistocardiography (BCG) signals, for BP prediction. While these PTT-based methods show some effectiveness, the linear relationship between the extracted features and BP limits their ability to capture complex BP variations. In contrast, studies based on PWA technology have focused on uncovering the nonlinear relationships between features and BP. Researchers like Yousefian et al. [23] and Miao et al. [39] have analyzed PWA features from multiple signals and applied nonlinear algorithms for BP prediction, achieving relatively good results. However, these methods still leave room for performance improvement.

As shown in Table 3, the system proposed in this study significantly outperforms other methods in terms of BP prediction accuracy. By integrating ECG, PPG, and ICG signals and combining traditional features with PWA features, the BP prediction performance can be effectively improved. Specifically, the MAE for SBP and DBP were 3.76 ± 3.98 mmHg and 2.71 ± 2.57 mmHg, respectively, achieving an A-grade performance under the BHS standard. These results demonstrate that the multi-signal-based BP monitoring system proposed in this study offers notable advantages in both accuracy and reliability, showcasing its strong potential for practical applications.

Compared with previous studies, our method offers two key advantages. First, it incorporates ICG signals alongside ECG and PPG signals. While ECG and PPG are commonly used to estimate BP through PAT or PTT, they are limited in their ability to capture hemodynamic parameters closely associated with BP, such as stroke volume and cardiac output [41,42,43]. Second, our approach employs a PWA method to comprehensively extract and analyze signal features. These two advantages complement each other to enhance estimation accuracy. Using the PWA method, we extract time intervals between the R peak of the ECG signal and the B, C, and X points of the ICG signal. These intervals correspond to different phases of cardiac contraction and are indicative of cardiac output. Additionally, the amplitudes of points B and C—and their difference—provide further physiological insights [44,45]. A lower B-point amplitude suggests more pronounced diastolic filling, while a higher C-point amplitude indicates stronger ventricular ejection. The amplitude ratio between these two points is correlated with stroke volume. By integrating these informative features from ICG signals using PWA, our model can capture subtle variations in BP that may be overlooked by ECG–PPG-based methods alone, thereby improving prediction accuracy.

### 5.4. Comparison with Existing Systems

To further clarify the practical value of the proposed system, we compared it with commercial BP monitoring devices and representative research prototypes. Table 4 summarizes the advantages and disadvantages of different systems, including commercial PPG-based wearable devices, commercial ICG monitors, ECG-PPG systems, multi-PPG systems, and the proposed ECG-PPG-ICG system.

Commercial PPG-based wearable devices (such as smartwatches) are compact and convenient for daily use, but their accuracy is limited by susceptibility to motion artifacts and a lack of direct hemodynamic information. Commercial ICG devices can reliably measure stroke volume and cardiac output, but they are bulky and unsuitable for daily monitoring. ECG-PPG systems have been widely studied to estimate BP using features such as PAT and PTT; however, their reliance on linear assumptions and lack of hemodynamic parameters limit their predictive performance. Multi-PPG methods aim to improve accuracy by utilizing multiple wavelengths or measurement points, but they still face challenges in capturing dynamic cardiac function, which is directly related to BP regulation.

The proposed ECG-PPG-ICG system integrates the complementary information of three signals into a compact acquisition platform. This design not only enhances feature richness by combining temporal and morphological parameters of ECG, PPG, and ICG but also retains the potential for miniaturization and wearable applications. Compared with existing research and commercial systems, our system achieves the accuracy of BP prediction while maintaining the practicality of continuous monitoring.

### 5.5. Limitations and Future Works

Although this study has validated the feasibility of a continuous BP monitoring system based on ECG, PPG, and ICG signals, there are still some limitations that need to be addressed for further optimization and enhancement of its practical applicability. First, ECG, PPG, and ICG signals are susceptible to motion artifacts. For instance, walking can introduce noise, hinder feature extraction, and reduce monitoring accuracy. Second, the system currently relies on USB power and lacks an independent power source, necessitating the development of a self-sustained power supply for wearable integration. Third, the study included only 40 young healthy participants (average age 23.9 ± 1.3 years), limiting the generalizability of the findings. In particular, the system’s performance in middle-aged or elderly populations, hypertensive individuals, and those with arrhythmias remains untested. Finally, all measurements in this study were conducted in the sitting position only. Body posture, such as lying down or standing, can alter cardiovascular dynamics and thus affect the accuracy of blood pressure estimation. Future work will focus on addressing these limitations to enhance the system’s practicality and robustness in real-world applications. First, advanced signal processing techniques and noise reduction algorithms will be developed to minimize the impact of motion artifacts on ECG, PPG, and ICG signals, ensuring reliable feature extraction and monitoring accuracy. Second, the hardware design will be further optimized by incorporating independent power modules and low-power controllers to reduce resource consumption, making the system more suitable for wearable devices. Third, the system will be validated on a larger and more diverse population, including individuals with varying health conditions, to improve its generalizability and clinical applicability. Finally, future experiments will include multiple body positions to evaluate system robustness across different physiological states. In conclusion, by addressing these limitations through future optimization and advancements, this system has the potential to become a more practical and reliable solution for non-invasive, convenient, and accurate continuous BP monitoring.

## 6. Conclusions

This study presents a continuous BP monitoring system that integrates multiple signals. The system simultaneously acquires and processes ECG, PPG, and ICG signals, extracting both traditional BP-related features (such as PAT and PTT) and PWA features from PPG and ICG. To evaluate the system’s effectiveness, a series of experiments were conducted comparing the predictive performance of different feature sets across various machine learning models. The results demonstrate that the fusion of multiple PWA features combined with the XGBoost model delivers the best BP prediction performance. The MAE for SBP is 3.76 ± 3.98 mmHg, and for DBP, it is 2.71 ± 2.57 mmHg, both of which achieve Grade A performance according to the BHS standard. The results demonstrate the significant advantages of the proposed multi-signal-based BP monitoring system. By leveraging the complementary strengths of multiple physiological signals and machine learning algorithms, the system delivers a highly accurate, reliable, and practical solution for continuous BP monitoring. Future work will involve large-scale validation across a broader population.

## Figures and Tables

**Table 1:** Performance evaluation of SBP and DBP prediction values.

Feature	BP	MAE	ME	SDE	PCC
PAT	SBP	5.75	2.27	4.75	0.81
	DBP	3.92	−0.30	2.91	0.61
PTT	SBP	8.27	3.01	7.70	0.23
	DBP	4.18	0.45	3.53	0.14
PPG-PWA	SBP	3.95	1.48	4.47	0.80
	DBP	2.76	−0.27	2.71	0.66
ICG-PWA	SBP	4.22	1.46	4.29	0.80
	DBP	3.04	0.06	2.76	0.61
MS-PWA	SBP	3.76	1.21	3.98	0.85
	DBP	2.71	−0.20	2.57	0.69

**Table 2:** Comparison between our results and BHS standard.

Comparison		Cumulative Error Percentage		
		≤5 mmHg	≤10 mmHg	≤15 mmHg
MS-PWA Model	SBP	71.52%	89.32%	96.89%
	DBP	81.97%	96.59%	99.55%
BHS standard	Grade A	60%	85%	95%
	Grade B	50%	75%	90%
	Grade C	40%	65%	85%

**Table 3:** Comparison of our proposed method with compared works.

Related Works	Signals	SBP (MAE ± SDE)	DBP (MAE ± SDE)
Yousefian et al. [23]	PPG, BCG, ECG	7.20 ± 0.90	4.70 ± 0.50
Ganti et al. [37]	PPG, SCG, ECG	4.75 ± 2.29	2.72 ± 0.75
Kim et al. [38]	PPG, BCG, ECG	7.30 ± 0.60	5.70 ± 0.40
Miao et al. [39]	ECG, 2-channel PPWs	6.13 ± 7.76	4.54 ± 5.52
Rachim et al. [40]	PPG, IPG	6.86 ± 1.65	6.67 ± 1.75
This work	ECG, ICG, PPG	3.76 ± 3.98	2.71 ± 2.57

**Table 4:** Comparison of representative BP monitoring systems.

System Type	Signals Used	Advantages	Disadvantages
Commercial PPG-only wearables (e.g., smartwatches)	PPG	Compact, user-friendly, already widely adopted; suitable for daily use	Limited accuracy due to motion artifacts; performance degrades in diverse populations
Commercial clinical ICG monitors (e.g., NICOM)	ICG	Accurate cardiac output and stroke volume assessment	Bulky; unsuitable for continuous daily monitoring
Research ECG–PPG systems	ECG, PPG	Well-established PAT/PTT features; simpler hardware	Cannot capture detailed hemodynamic changes; limited accuracy in BP variation tracking
Research multi-PPG systems	PPG × N	Improve robustness by exploiting differential timing and absorption	Accuracy affected by peripheral circulation
Proposed system	ECG, PPG, ICG	Integrates multiple signals in a compact platform; provides hemodynamic parameters; potential for wearable integration	Requires hardware miniaturization and larger-scale validation

## Data Availability

Please contact the corresponding author to request access to the data mentioned in this article, but note that it cannot be used for commercial activities.

## Supplementary Materials

The following supporting information can be downloaded at https://www.mdpi.com/article/10.3390/s25185910/s1, Table S1: PPG features for estimating SBP after feature selection. Table S2: PPG features for estimating DBP after feature selection. Table S3: ICG features for estimating SBP after feature selection. Table S4: ICG features for estimating DBP after feature selection. Table S5: Multi-signal features for estimating SBP after feature selection. Table S6: Multi-signal features for estimating DBP after feature selection. Table S7: List of features extracted from the PPG. Table S8: List of features extracted from the ICG.

## Appendix A

Table A1 presents the performance evaluation of SBP and DBP prediction using five-fold cross-validation.

**Table A1:** Performance evaluation of SBP and DBP prediction values in five-fold cross-validation.

Feature	BP	MAE	ME	SDE	PCC
PAT	SBP	11.11	0.02	13.48	0.40
	DBP	6.47	0.04	8.01	0.27
PTT	SBP	11.76	−0.00	14.9	−0.24
	DBP	6.67	0.00	8.32	−0.11
PPG-PWA	SBP	8.70	0.22	11.09	0.65
	DBP	5.89	0.07	7.36	0.46
ICG-PWA	SBP	9.39	−0.01	12.17	0.56
	DBP	6.61	0.40	8.22	0.14
MS-PWA	SBP	8.06	0.64	10.59	0.69
	DBP	5.68	0.14	7.22	0.49
