# Supplementary material for: Multi-Signal Acquisition System for Continuous Blood Pressure Monitoring
Source: Sensors (Basel). 2025 Sep 21;25(18):5910. doi: 10.3390/s25185910 (PMC12473825; doi:10.3390/s25185910)
Supplement: Supplementary file 1 [file sensors-25-05910-s001.zip › sensors-3857463-supplementary.pdf]

contents

Supplementary materials 2

Supplementary material S1 2

Supplementary material S2 4

Supplementary material S3 5

Supplementary material S4 6

Supplementary material S5 7

Supplementary material S6 12

Supplementary material S7 15

Supplementary materials

Supplementary material S1

The PPG signal is first generated by the photodiode in the pulse oximeter finger clip, which converts the light intensity into a current in the nanoampere to microampere range. Then, the current is converted into a voltage signal by a current-to-voltage (I/V) conversion circuit, the key circuit of which is shown in Fig 1. Specifically, the cathode of the photodiode is connected to the inverting input of the differential amplifier, and the positive is connected to the non-inverting input. A 510 kΩ feedback resistor and a 100 kΩ input resistor are used, resulting in a gain of 5.1 (510/100). The selection of a 510 kΩ feedback resistor can effectively convert the weak photocurrent into a voltage signal in the millivolt range for further processing. In the primary amplification stage, the signal is amplified with a gain of 8 to improve the signal-to-noise ratio while avoiding excessive noise amplification. To suppress baseline drift (mainly caused by breathing or movement), an active second-order Butterworth high-pass filter with a cutoff frequency of 0.05 Hz and a gain of 1.6 is used. This is followed by an active second-order Butterworth low-pass filter with a cutoff frequency of 10 Hz and a gain of 1.6 to remove high-frequency noise such as harmonics generated by power line interference. Together, these filters form a fourth-order bandpass filter that preserves the main frequency components of the PPG signal, typically in the range of 0.5–4 Hz (heart rate). To maintain a balance between passband flatness and roll-off steepness, the Butterworth filter uses a cascaded second-order configuration. Finally, the signal passes through a non-inverting amplifier stage (gain 2) to fine-tune the amplitude, and then the DC bias is shifted to 1.65 V to ensure compatibility with the single-supply input range of the ADC.

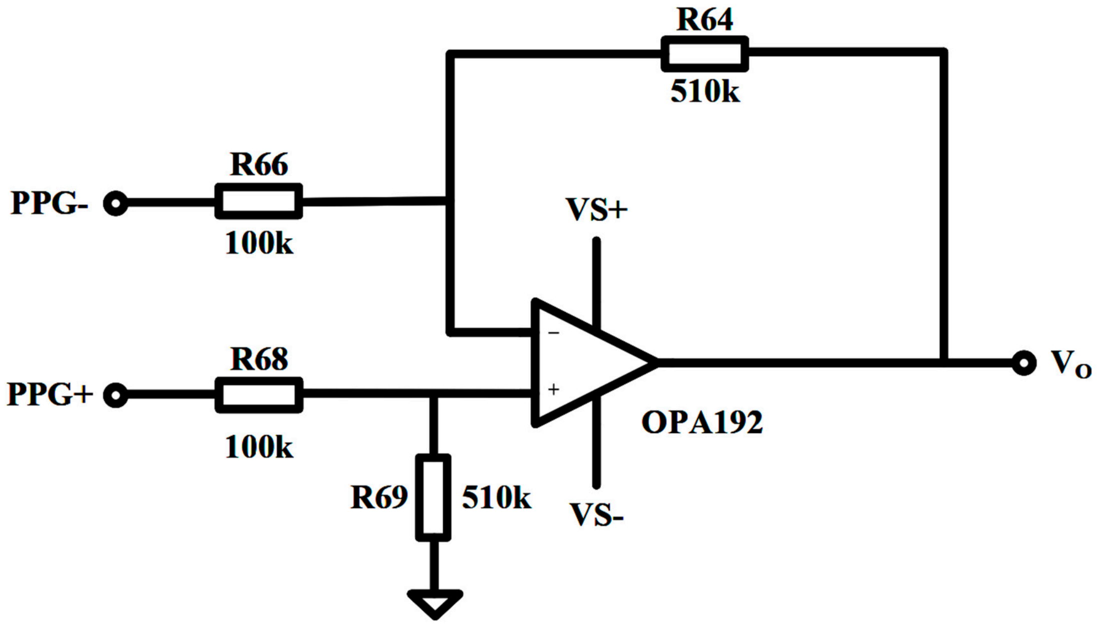

Figure S1. Transimpedance amplifier circuit

The ECG signal is acquired using a standard Lead II configuration. The resulting differential signal (ranging from 0.1 mV to 5 mV) is first converted into a single-ended signal

by an INA828 instrumentation amplifier, chosen for its high common-mode rejection ratio (CMRR) and low noise. The amplifier is configured with a gain of 11, selected to balance signal amplitude with the needs of subsequent amplification stages. To remove baseline drift—typically introduced by respiration or unstable electrode contact—an active second-order Butterworth high-pass filter with a cutoff frequency of 0.5 Hz and a gain of 1.6 is applied. The signal is then amplified in a non-inverting configuration with a gain of 6 to increase its amplitude. High-frequency noise, including powerline harmonics and electromyographic (EMG) interference, is attenuated using an active second-order Butterworth low-pass filter with a cutoff frequency of 100 Hz and a gain of 1.6. The cascaded second-order Butterworth filter design ensures a balanced trade-off between passband flatness and sharp frequency roll-off. The final stage consists of another non-inverting amplifier (gain 6) to adjust the signal amplitude to match the ADC input range. A DC bias of 1.65 V is added to shift the signal into the single-supply input range of the ADC. To further suppress powerline interference (50/60 Hz) and enhance CMRR, a right-leg drive (RLD) circuit is implemented, the key circuit of which is shown in Fig 2. The common-mode voltage is obtained by averaging the voltages at the positive and negative input electrodes, buffered by a voltage follower, and fed into an inverting amplifier with a gain of 40. The output is then returned to the body through the RLD electrode, forming a negative feedback loop that significantly improves interference rejection. The gain of 40 is selected based on the expected amplitude of the common-mode signal (from millivolts to volts) and the drive capability required by the RLD circuit.

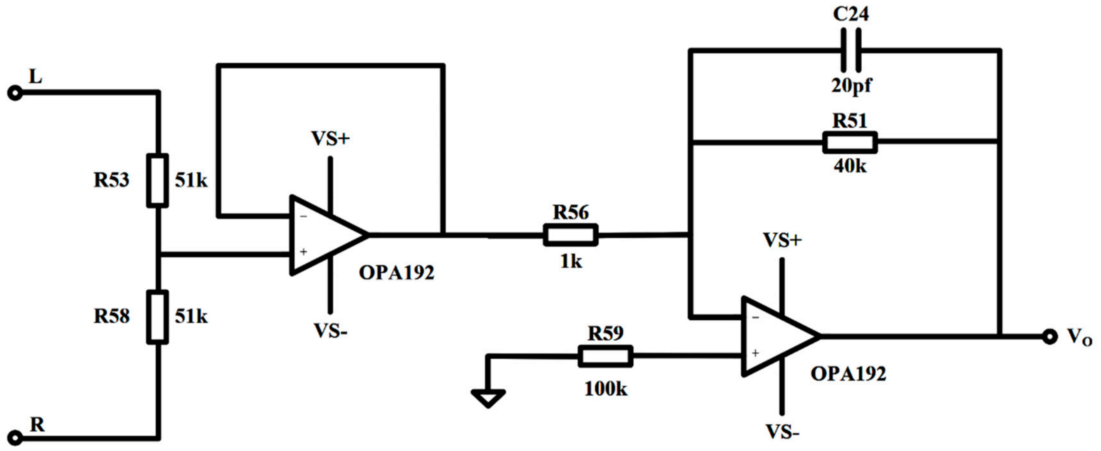

Figure S2. Right-leg drive circuit

Supplementary material S2

ECG, PPG, and ICG signals are susceptible to a variety of noise disturbances in wearable devices: motion artifacts are mainly caused by the relative displacement of the electrodes to the skin or muscle activity, which may manifest as baseline drift or pseudo-QRS waves in ECG, cause signal distortion in PPG, and affect the stability of the impedance baseline in ICG; Powerline disturbances (50/60 Hz) are introduced through electromagnetic coupling and mainly affect the high-frequency components of the ECG and ICG.

In response to these noises, we have adopted the following mitigation strategies:

Firstly, at the hardware level, we use shielding technology to reduce the electromagnetic coupling of power line interference by using shielding and grounding design around the signal acquisition circuit, and at the same time optimize the electrode design (such as using highly conductive gel electrodes) to reduce the electrode-skin contact impedance and reduce the effect of motion artifacts.

Secondly, at the signal processing level, the active second-order Butterworth high-pass filter (cut-off frequency 0.5 Hz) is used to suppress the baseline drift in the ECG signal chain, the low-pass filter (cut-off frequency is 100 Hz) to filter out the high-frequency noise, and the common-mode rejection ratio (CMRR) is increased in combination with the right-leg drive (RLD) circuit (gain 40), which effectively cancels the power line interference. The PPG signal is filtered out by cascading second-order Butterworth high-pass (0.05 Hz) and low-pass (10 Hz) filters, and digital filtering is introduced at the algorithm level to further eliminate the interference of motion artifacts on the pulse wave morphology. For ICG signals, the baseline drift is removed by high-pass filtering (0.16 Hz), high-pass filtering (8 Hz) is used to suppress high-frequency noise, and differential measurement techniques are used to reduce common-mode interference.

In addition, in the system design, we optimized the fit of the wearable device to reduce the occurrence of motion artifacts through flexible materials and structural design that adheres to the skin.

Supplementary material S3

The system acquires PPG, ECG, and ICG signals simultaneously via ADS1256, and to ensure data integrity and minimize inter-module interference, we have taken the following measures.

During the signal acquisition phase, three signals—ICG (connected to AIN0), ECG (AIN3), and PPG (AIN4)—were synchronously collected using the ADS1256 analog-to-digital converter at a sampling rate of 250 Hz. Acquisition was triggered by the STM32F103RCT6 timer and executed via the ADS1256's built-in multiplexer, internal 8 MHz crystal oscillator, and the SYNC/PDWN synchronization control pin. This configuration ensured precise channel switching and sampling timing, thereby minimizing inter-module timing deviations and maintaining data synchronization across all signals.

In terms of hardware design, to minimize signal crosstalk in compact wearable systems, the front-end signal conditioning circuits—including differential amplification, filtering, and baseline elevation—for PPG, ECG, and ICG were modularized and physically separated on the PCB by at least 10 mm. This layout reduces capacitive coupling and electromagnetic interference. Each module operates within its own power domain (AVDD at 5 V and DVDD at 3.3 V), with decoupling capacitors (100 nF and 10 μF) employed to stabilize power supply. Additionally, analog signal paths (e.g., PPG photodiode, ECG electrodes, and ICG impedance electrodes) were isolated from digital signal paths (e.g., SPI lines) using dedicated ground planes in the PCB layout. A 100 Ω resistor was placed near the ADS1256 input pins to achieve signal impedance matching and further suppress high-frequency crosstalk.

At the software level, upon receiving SPI data from the ADS1256, the microcontroller assembles the PPG, ECG, and ICG signals into ordered data frames (ICG, ECG, PPG). These frames are then transmitted to a PC via serial communication at a baud rate of 115200 bps. The resulting data rate of 18 kbps (250 Hz × 9 bytes) remains well below the bandwidth limit of the serial interface, thereby ensuring reliable data transmission.

Supplementary material S4

The system simultaneously acquires ECG, PPG, and ICG signals via ADS1256, ADS1256 transmits them to the microcontroller STM32F103RCT6 via the SPI protocol using an 8 MHz crystal oscillator. The MCU uses a timer to trigger ADS1256 at a frequency of 250 Hz to synchronously acquire three signals (i.e., a sampling rate of 250 Hz, which meets the bandwidth requirements of 0.5 Hz to 100 Hz for ECG signals, 0.05 Hz to 10 Hz for PPG signals, and 0.5 Hz to 30 Hz for ICG signals), and then transmits them to the MCU via the SPI protocol, where they are framed in the MCU and transmitted to the PC via the serial port. ADS1256 24-bit resolution, 24 bits (3 bytes) per sample per channel, and a total data rate of 250 Hz for three channels (ECG, PPG, ICG) × 3 bytes for 3 channels × = 2.25 kB/s (18 kbps).

The SPI protocol runs at a clock frequency of 10 MHz (configured by STM32F103RCT6), and considering the transmission overhead (about 10%), the actual throughput can reach 9 Mbps, which is much higher than the 18 kbps requirement, so there is no bottleneck in SPI transmission. The internal processing capacity of the MCU (STM32F103RCT6, 72 MHz main frequency) is sufficient to support data framing operations, and the data is transmitted to the PC through the serial port (baud rate 115200 bps) after framing, and the serial port bandwidth is higher than 18 kbps to meet the real-time transmission requirements.

To avoid potential bottlenecks, we implemented a data buffering mechanism (using a 512-byte ring buffer) in the MCU to ensure that the data stream collected by the ADS1256 is not lost during SPI transmission and serial transmission. In addition, the power supply design of ADS1256 in the circuit diagram (AVDD 5 V, DVDD 3.3 V with 100 nF and 10 μF decoupling capacitors) and the reference voltage pins VREFP and VREFN (stabilized by 100 nF and 47 μF filter capacitors) ensure stable signal sampling.

Supplementary material S5

Although the features extracted from PPG and ICG signals contain valuable information related to blood pressure variations, the original feature set may include redundant or irrelevant features. Using all features directly in model construction can degrade model performance. Feature selection is therefore a crucial step in machine learning workflows, as it can significantly enhance both model efficiency and accuracy. In this study, we employed the Least Absolute Shrinkage and Selection Operator (LASSO) regression algorithm for feature selection. The regularization hyperparameter α was set to 0.1, a commonly used value in similar applications. LASSO introduces an L1-norm penalty to the regression model, which not only minimizes prediction error but also performs variable selection by shrinking some coefficients to zero. This allows for effective feature filtering and model simplification. Using LASSO, we retained only the features most relevant to BP. The following 6 tables respectively show the features retained after feature selection using SBP and DBP estimated from PPG, ICG and multi-signal features.

Table S1. PPG features for estimating SBP after feature selection

Number	Symbols	Definition
1	PTT2	The time from the ECG signal R point to the first-order derivative peak point of the PPG signal
2	max_min_Amp_rate_d	min_Point_dPPG_Amp/max_Point_dPPG_Amp
3	sd1_sd2	Ratio of sd1 to sd2
4	breathingrate	Respiratory rate
5	Freq_3	Peak_3 corresponding frequency
6	dia_len_10_Amp_rate	dia_len_10_Amp/P_Amp
7	max_min_time_rate_dd	min_Point_ddPPG_time/max_Point_ddPPG_time
8	pnn50	The percentage of adjacent heartbeat intervals greater than 50ms
9	hr_mad	Median absolute deviation of heart rate
10	Freq_1	Peak_1 corresponding frequency
11	pnn20	The percentage of adjacent heartbeat intervals greater than 20ms
12	dia_area_rate	dia_area/PPG_area
13	sys_area_rate	sys_area/PPG_area
14	min_Point_dPPG_Amp	Amplitude of the minimum first-order derivative
15	S_kurtosis	The kurtosis of the signal
16	S_median	The median value of the signal
17	Freq_2	Peak_2 corresponding frequency
18	S_mean	The mean value of the signal
19	PTT1	The time from the ECG signal R point to the PPG signal peak point
20	r_p12	Peak_1/Peak_2
21	max_Point_dPPG_Amp	Amplitude of the maximum first-order derivative

Table S2. PPG features for estimating DBP after feature selection

Number	Symbols	Definition
1	PTT2	The time from the ECG signal R point to the first-order derivative peak point of the PPG signal
2	breathingrate	Respiratory rate
3	max_min_Amp_rate_d	min_Point_dPPG_Amp/max_Point_dPPG_Amp
4	pnn20	The percentage of adjacent heartbeat intervals greater than 20ms
5	pnn50	The percentage of adjacent heartbeat intervals greater than 50ms
6	sd1_sd2	Ratio of sd1 to sd2
7	dia_len_10_Amp_rate	dia_len_10_Amp/P_Amp
8	dia_area_rate	dia_area/PPG_area
9	max_min_time_rate_dd	min_Point_ddPPG_time/max_Point_ddPPG_time
10	Freq_3	Peak_3 corresponding frequency
11	S_mean	The mean value of the signal
12	min_Point_dPPG_Amp	Amplitude of the minimum first-order derivative
13	sys_area_rate	sys_area/PPG_area

Table S3. ICG features for estimating SBP after feature selection

Number	Symbols	Definition
1	R_cb	Ratio of amplitudes at points C and B
2	breathingrate	Respiratory rate
3	R_xb	Ratio of amplitudes at points X and B
4	H_cb_25	25% of the amplitude difference between point C and B
5	S_mean	The mean value of the signal
6	pnn50	The percentage of adjacent heartbeat intervals greater than 50ms
7	Freq_3	Peak_3 corresponding frequency
8	S_median	The median value of the signal
9	Freq_1	Peak_1 corresponding frequency
10	sd1_sd2	Ratio of sd1 to sd2
11	Freq_2	Peak_2 corresponding frequency
12	pnn20	The percentage of adjacent heartbeat intervals greater than 20ms
13	R_bx	Ratio of amplitudes at points B and X
14	hr_mad	Median absolute deviation of heart rate
15	TR_s_d	Ratio of systolic to diastolic period
16	T_rc	Time difference between points R and C
17	D_bc_max	The maximum value of the derivative between point C and B

18	r_p13	Peak_1/Peak_3
19	sdsd	The standard deviation of the difference between adjacent heartbeats
20	T_rb	Time difference between points R and B
21	D_cx_max	The maximum value of the derivative between point C and X
22	Peak_2	The second largest peak of the ICG signal in the FFT amplitude spectrum
23	H_cb_75	75% of the amplitude difference between point C and B
24	r_p12	Peak_1/Peak_2
25	D_bc_min	The minimum value of the derivative between point C and B
26	T_bc	Time difference between points B and C
27	T_rx	Time difference between points R and X
28	S_kurtosis	The kurtosis of the signal

Table S4. ICG features for estimating DBP after feature selection

Number	Symbols	Definition
1	R_cb	Ratio of amplitudes at points C and B
2	R_xb	Ratio of amplitudes at points X and B
3	breathingrate	Respiratory rate
4	S_median	The median value of the signal
5	pnn50	The percentage of adjacent heartbeat intervals greater than 50ms
6	sd1_sd2	Ratio of sd1 to sd2
7	pnn20	The percentage of adjacent heartbeat intervals greater than 20ms
8	T_rc	Time difference between points R and C
9	D_cx_max	The maximum value of the derivative between point C and X
10	Freq_3	Peak_3 corresponding frequency
11	TR_s_d	Ratio of systolic to diastolic period
12	Amp_b	Amplitude at point B
13	R_bx	Ratio of amplitudes at points B and X
14	Freq_1	Peak_1 corresponding frequency
15	S_mean	The mean value of the signal
16	H_cb_50	50% of the amplitude difference between point C and B
17	H_cb_75	75% of the amplitude difference between point C and B
18	H_cb_25	25% of the amplitude difference between point C and B

19	Freq_2	Peak_2 corresponding frequency
20	T_rb	Time difference between points R and B
21	sdsd	The standard deviation of the difference between adjacent heartbeats
22	hr_mad	Median absolute deviation of heart rate
23	S_kurtosis	The kurtosis of the signal
24	T_bc	Time difference between points B and C
25	r_p13	Peak_1/Peak_3

Table S5. Multi-signal features for estimating SBP after feature selection

Number	Symbols	Definition
1	max_min_Amp_rate_d	min_Point_dPPG_Amp/max_Point_dPPG_Amp
2	PTT1	The time from the ECG signal R point to the PPG signal peak point
3	PTT2	The time from the ECG signal R point to the first-order derivative peak point of the PPG signal
4	pnn20	The percentage of adjacent heartbeat intervals greater than 20ms
5	dia_len_10_Amp_rate	dia_len_10_Amp/P_Amp
6	breathingrate	Respiratory rate
7	max_min_time_rate_dd	min_Point_ddPPG_time/max_Point_ddPPG_time
8	S_mean	The mean value of the signal
9	Freq_3	Peak_3 corresponding frequency
10	sys_area_rate	sys_area/PPG_area
11	dia_area_rate	dia_area/PPG_area
**12**	**R_cb**	**Ratio of amplitudes at points C and B**
**13**	**S_mean**	**The mean value of the signal**
**14**	**T_rb**	**Time difference between points R and B**
**15**	**H_cb_25**	**25% of the amplitude difference between point C and B**
**16**	**pnn20**	**The percentage of adjacent heartbeat intervals greater than 20ms**
**17**	**breathingrate**	**Respiratory rate**
**18**	**S_median**	**The median value of the signal**
**19**	**T_bc**	**Time difference between points B and C**
**20**	**Freq_1**	**Peak_1 corresponding frequency**
**21**	**T_rx**	**Time difference between points R and X**
**22**	**R_cb**	**Ratio of amplitudes at points C and B**
**23**	**Freq_3**	**Peak_3 corresponding frequency**
**24**	**H_cb_75**	**75% of the amplitude difference between point C and B**

The non-bold ones are features extracted from the PPG signal, and the bold ones are extracted from the ICG signal.

Table S6. Multi-signal features for estimating DBP after feature selection

Number	Symbols	Definition
1	PTT2	The time from the ECG signal R point to the first-order derivative peak point of the PPG signal
2	max_min_Amp_rate_d	min_Point_dPPG_Amp/max_Point_dPPG_Amp
3	pnn50	The percentage of adjacent heartbeat intervals greater than 50ms
4	breathingrate	Respiratory rate
5	dia_len_10_Amp_rate	dia_len_10_Amp/P_Amp
**6**	**S_mean**	**The mean value of the signal**
**7**	**R_cb**	**Ratio of amplitudes at points C and B**
**8**	**R_xb**	**Ratio of amplitudes at points X and B**
**9**	**pnn20**	**The percentage of adjacent heartbeat intervals greater than 20ms**

The non-bold ones are features extracted from the PPG signal, and the bold ones are extracted from the ICG signal.

Supplementary material S6

Table S7. List of features extracted from the PPG

Number	Symbols	Definition
1	PTT1	The time from the ECG signal R point to the PPG signal peak point
2	PTT2	The time from the ECG signal R point to the first-order derivative peak point of the PPG signal
3	S_Amp	Amplitude of trough
4	P_Amp	The peak amplitude
5	P_S_Height	The difference between the amplitude at point P and S
6	sys_len_10_Amp	Amplitude at 10% length of S-P segment
7	sys_len_25_Amp	Amplitude at 25% length of S-P segment
8	sys_len_33_Amp	Amplitude at 33% length of S-P segment
9	sys_len_50_Amp	Amplitude at 50% length of S-P segment
10	sys_len_66_Amp	Amplitude at 66% length of S-P segment
11	sys_len_75_Amp	Amplitude at 75% length of S-P segment
12	dia_len_10_Amp	Amplitude at 10% length of P-S (next cardiac cycle) segment
13	dia_len_25_Amp	Amplitude at 25% length of P-S (next cardiac cycle) segment
14	dia_len_33_Amp	Amplitude at 33% length of P-S (next cardiac cycle) segment
15	dia_len_50_Amp	Amplitude at 50% length of P-S (next cardiac cycle) segment
16	dia_len_66_Amp	Amplitude at 66% length of P-S (next cardiac cycle) segment
17	dia_len_75_Amp	Amplitude at 75% length of P-S (next cardiac cycle) segment
18	sys_len_10_Amp_rate	sys_len_10_Amp/P_Amp
19	sys_len_25_Amp_rate	sys_len_25_Amp/P_Amp
20	sys_len_33_Amp_rate	sys_len_33_Amp/P_Amp
21	sys_len_50_Amp_rate	sys_len_50_Amp/P_Amp
22	sys_len_66_Amp_rate	sys_len_66_Amp/P_Amp
23	sys_len_75_Amp_rate	sys_len_75_Amp/P_Amp
24	dia_len_10_Amp_rate	dia_len_10_Amp/P_Amp
25	dia_len_25_Amp_rate	dia_len_25_Amp/P_Amp
26	dia_len_33_Amp_rate	dia_len_33_Amp/P_Amp
27	dia_len_50_Amp_rate	dia_len_50_Amp/P_Amp
28	dia_len_66_Amp_rate	dia_len_66_Amp/P_Amp
29	dia_len_75_Amp_rate	dia_len_75_Amp/P_Amp
30	sys_time	The time from point S to P

31	dia_time	The time from point P to S of the next cardiac cycle
32	all_time	The duration of a cardiac cycle
33	sys_time_rate	sys_time/all_time
34	dia_time_rate	dia_time/all_time
35	PPG_area	The area of a PPG signal segment in one cycle
36	sys_area	Area of S-P segment
37	dia_area	Area of P-S (next cardiac cycle) segment
38	sys_area_rate	sys_area/PPG_area
39	dia_area_rate	dia_area/PPG_area
40	PS_K	The slope between point S and point P
41	S_mean	The mean value of the signal
42	S_std	The standard deviation of the signal
43	S_var	The variance of the signal
44	S_max	The maximum value of the signal
45	S_min	The minimum value of the signal
46	S_range	The difference between the maximum and minimum values of the signal
47	S_median	The median value of the signal
48	S_skew	The skewness of the signal
49	S_kurtosis	The kurtosis of the signal
50	max_Point_dPPG_time	Time of maximum first-order derivative
51	max_Point_dPPG_Amp	Amplitude of the maximum first-order derivative
52	min_Point_dPPG_time	Time of minimum first-order derivative
53	min_Point_dPPG_Amp	Amplitude of the minimum first-order derivative
54	max_min_time_rate_d	min_Point_dPPG_time/max_Point_dPPG_time
55	max_min_Amp_rate_d	min_Point_dPPG_Amp/max_Point_dPPG_Amp
56	max_Point_ddPPG_time	Time of maximum second-order derivative
57	max_Point_ddPPG_Amp	Amplitude of the maximum second -order derivative
58	min_Point_ddPPG_time	Time of minimum second -order derivative
59	min_Point_ddPPG_Amp	Amplitude of the minimum second -order derivative
60	max_min_time_rate_dd	min_Point_ddPPG_time/max_Point_ddPPG_time
61	max_min_Amp_rate_dd	min_Point_ddPPG_Amp/max_Point_ddPPG_Amp
62	bpm	Beats per minute
63	ibi	The average time interval between consecutive heartbeats
64	sdnn	The standard deviation of all normal heartbeat intervals (NN intervals)
65	sdsd	The standard deviation of the difference between adjacent heartbeats
66	rmssd	The square root of the difference between adjacent heart beats
67	pnn20	The percentage of adjacent heartbeat intervals greater than 20ms

68	pnn50	The percentage of adjacent heartbeat intervals greater than 50ms
69	hr_mad	Median absolute deviation of heart rate
70	sd1	Poincaré plot short-term variability
71	sd2	Poincaré plot long-term variability
72	s	Pi* sd1* sd2
73	sd1_sd2	Ratio of sd1 to sd2
74	breathingrate	Respiratory rate
75	Peak_1	The maximum peak of the PPG signal in the FFT amplitude spectrum
76	Peak_2	The second largest peak of the PPG signal in the FFT amplitude spectrum
77	Peak_3	The third largest peak of the PPG signal in the FFT amplitude spectrum
78	Freq_1	Peak_1 corresponding frequency
79	Freq_2	Peak_2 corresponding frequency
80	Freq_3	Peak_3 corresponding frequency
81	r_p12	Peak_1/Peak_2
82	r_p13	Peak_1/Peak_3
83	r_f12	Freq_1/Freq_2
84	r_f13	Freq_1/Freq_3
85	total_power	The total energy of the entire spectrum
86	max_power	The frequency component with the highest energy in the spectrum
87	mean_power	The average value of the energy of all frequency components
88	spectral_entropy	Spectral entropy

Supplementary material S7

Table S8. List of features extracted from the ICG

Number	Symbols	Definition
1	T_rb	Time difference between points R and B
2	T_rc	Time difference between points R and C
3	T_rx	Time difference between points R and X
4	T_bc	Time difference between points B and C
5	T_cx	Time difference between points C and X
6	TR_bc	Ratio of the time between points B and C to the duration of the cardiac cycle
7	TR_cx	Ratio of the time between points C and X to the duration of the cardiac cycle
8	TR_bx	Ratio of the time between points B and X to the duration of the cardiac cycle
9	TR_s_d	Ratio of systolic to diastolic period
10	Amp_b	Amplitude at point B
11	Amp_c	Amplitude at point C
12	Amp_x	Amplitude at point X
13	H_cx	Amplitude difference between points C and X
14	H_cb	Amplitude difference between points C and B
15	H_cb_25	25% of the amplitude difference between point C and B
16	H_cb_50	50% of the amplitude difference between point C and B
17	H_cb_75	75% of the amplitude difference between point C and B
18	D_bc_max	The maximum value of the derivative between point C and B
19	D_bc_min	The minimum value of the derivative between point C and B
20	D_cx_max	The maximum value of the derivative between point C and X
21	D_cx_min	The minimum value of the derivative between point C and X
22	R_bc	Ratio of amplitudes at points B and C
23	R_bx	Ratio of amplitudes at points B and X
24	R_cx	Ratio of amplitudes at points C and X
25	R_cb	Ratio of amplitudes at points C and B
26	R_xb	Ratio of amplitudes at points X and B
27	R_xc	Ratio of amplitudes at points X and C
28	K_bc	Slope between B and C points
29	K_cx	Slope between C and X points

30	bcx_area	Area enclosed by the BCX wave
31	S_mean	The mean value of the signal
32	S_std	The standard deviation of the signal
33	S_var	The variance of the signal
34	S_max	The maximum value of the signal
35	S_min	The minimum value of the signal
36	S_range	The difference between the maximum and minimum values of the signal
37	S_median	The median value of the signal
38	S_skew	The skewness of the signal
39	S_kurtosis	The kurtosis of the signal
40	bpm	Beats per minute
41	ibi	The average time interval between consecutive heartbeats
42	sdnn	The standard deviation of all normal heartbeat intervals (NN intervals)
43	sdsd	The standard deviation of the difference between adjacent heartbeats
44	rmssd	The square root of the difference between adjacent heart beats
45	pnn20	The percentage of adjacent heartbeat intervals greater than 20ms
46	pnn50	The percentage of adjacent heartbeat intervals greater than 50ms
47	hr_mad	Median absolute deviation of heart rate
48	sd1	Poincaré plot short-term variability
49	sd2	Poincaré plot long-term variability
50	s	Pi* sd1* sd2
51	sd1_sd2	Ratio of sd1 to sd2
52	breathingrate	Respiratory rate
53	Peak_1	The maximum peak of the ICG signal in the FFT amplitude spectrum
54	Peak_2	The second largest peak of the ICG signal in the FFT amplitude spectrum
55	Peak_3	The third largest peak of the ICG signal in the FFT amplitude spectrum
56	Freq_1	Peak_1 corresponding frequency
57	Freq_2	Peak_2 corresponding frequency
58	Freq_3	Peak_3 corresponding frequency
59	r_p12	Peak_1/Peak_2
60	r_p13	Peak_1/Peak_3
61	r_f12	Freq_1/Freq_2
62	r_f13	Freq_1/Freq_3

63	total_power	The total energy of the entire spectrum
64	max_power	The frequency component with the highest energy in the spectrum
65	mean_power	The average value of the energy of all frequency components
66	spectral_entropy	Spectral entropy
